# Immunological investigations of the cerebrospinal fluid in patients with recent onset psychotic disorders: A study protocol

**DOI:** 10.1371/journal.pone.0257946

**Published:** 2021-09-29

**Authors:** Rose Jeppesen, Sonja Orlovska-Waast, Nina Vindegaard Sørensen, Rune Haubo Bojesen Christensen, Michael Eriksen Benros

**Affiliations:** 1 Biological and Precision Psychiatry, Copenhagen Research Center for Mental Health, Mental Health Centre Copenhagen, Copenhagen University Hospital, Copenhagen, Denmark; 2 Department of Immunology and Microbiology, Faculty of Health and Medical Sciences, University of Copenhagen, Copenhagen, Denmark; Public Library of Science, UNITED STATES

## Abstract

**Background:**

Though many previous studies have indicated immunological alterations in psychotic disorders, the role and prevalence of neuroinflammation is still unknown. Studies previously investigating immune related biomarkers in the cerebrospinal fluid (CSF) of these patients are mainly small studies on few markers, and many have not compared patients to healthy controls.

**Methods:**

We will conduct a large case-control study including at least 100 patients with recent onset psychotic disorders and 100 sex- and age matched healthy controls. The cases will include patients diagnosed with a psychotic disorder according to ICD-10 (F20/F22-29) within a year prior to inclusion. We will collect both CSF, blood and fecal samples, to gain insight into possible immunological alterations. The psychopathology of all participants will thoroughly be evaluated using the SCAN interview, and multiple rating scales covering different symptom groups. All participants will partake in a detailed neurological examination, including the Neurological Evaluation Scale assessing neurological soft signs. Additionally, we will assess cognitive functioning, evaluate quality of life and level of functioning, and collect data on a broad array of possible confounders. Our primary outcomes will include CSF leucocytes, CSF/serum albumin ratio, CSF total protein, IgG index, CSF levels of IL-6 and IL-8, and presence of antineuronal autoantibodies in CSF and blood. For our secondary outcomes, exploratory analyses will be performed on a broader panel of neuroimmunological markers. All participants will be invited for a follow-up visit to assess longitudinal changes. The current study is part of a larger CSF biobank build-up for severe mental disorders (PSYCH-FLAME).

**Discussion:**

This study will represent the largest investigation of CSF in patients with psychotic disorders compared to healthy controls to date. We expect the study to contribute with new, important knowledge on pathophysiological mechanisms, and to help pave the way for future investigations of individualized treatment options.

**Trial registration:**

The study is approved by The Regional Committee on Health Research Ethics (Capital Region, j.no: H-16030985) and The Danish Data Protection Agency (j.no: RHP-2016-020, I-Suite no.: 04945).

## Background

Psychotic disorders are a heterogeneous group of mental disorders with massive impact on both the affected individuals, relatives and the society [1]. A substantial part of patients with psychotic disorders experience low treatment response [2, 3], and current antipsychotics have inadequate effects on the negative symptoms and cognitive deficits associated with schizophrenia [4–8], which impact functioning and quality of life [9, 10]. Standing in the way of improvement hereof, is the fact that the etiological mechanisms of psychotic disorders are, as of yet, poorly understood.

Mounting evidence now suggests involvement of inflammatory pathways in at least a subset of patients with psychotic disorders. Higher levels of pro-inflammatory cytokines in the blood of acutely ill patients with psychosis have been found [11], peripheral inflammation has been associated with greater severity of both cognitive deficits and negative symptoms [12], and elevated inflammatory biomarkers have been associated with lack of treatment response [13]. It has additionally been found, that autoimmune diseases, a group of diseases with clear inflammatory pathophysiologic mechanisms, increase the risk of psychotic disorders with as much as 45% [14], even when excluding neurological autoimmune diseases [15]. Furthermore, genome wide association studies have found associations between schizophrenia and genes in the HLA-region [16], a region responsible for encoding proteins involved in a wide array of immunological and inflammatory pathways and known to be involved in several autoimmune diseases. Building on the inflammatory hypothesis, anti-inflammatory add-on treatment has been investigated in patients with psychotic disorders. A recent meta-analysis [17] showed an overall effect of anti-inflammatory drugs on psychopathology including an effect on negative symptoms contrary to current antipsychotics.

Some studies have additionally found indications of neuroinflammation in the cerebrospinal fluid (CSF) of patients with psychotic disorders; a recent meta-analysis [18] showed increased levels of the pro-inflammatory cytokines interleukin 6 (IL-6) and IL-8 in the CSF, as well as increased CSF/serum albumin ratio, indicating increased blood-brain barrier permeability. Further adding to the immune hypothesis, psychotic symptoms can be induced by immune components such as antineuronal autoantibodies, as in anti-NMDA receptor autoimmune encephalitis, where 65% of the patients present with psychiatric symptoms, particularly psychosis, and 31% are initially admitted to a psychiatric ward [19], with immunomodulating treatment showing efficacy as the primary treatment [20].

However, the evidence on immunological alterations in CSF, and prevalence of immune components such as antineuronal antibodies in the CSF of patients with psychotic disorders, is still limited, and as CSF is the bodily fluid that can tell us the most about neuroinflammation, this knowledge is important to move further in the immunopsychiatric field. Most studies investigating immunological findings in the CSF of psychotic patients have assessed only single or few biomarkers of neuroinflammation, and while some cytokines such as IL-6 have been investigated in multiple studies, even when all available data hereon is combined in a meta-analysis, the total number of cases and healthy controls still do not exceed 250 [18]. The largest studies to date assessing inflammatory markers in the CSF of patients with psychosis compared to healthy controls, has been performed by a Japanese group [21, 22], comprising between 86 and 96 patients with schizophrenia and a group of matched healthy controls. However, they included only few clinical measurements and assessed only single biomarkers (matrix metalloproteinases and complement C5). Much of the other previously published studies on CSF include markedly smaller study samples, and many include no healthy controls as comparison. Additionally, many reports on previous CSF studies lack transparency regarding both laboratory and statistical analyses, lack information regarding the definition of their healthy controls and have not matched their cases and controls on important factors such as age and sex. Additionally, no studies to date have assessed biomarkers in the CSF of patients with psychotic disorders compared to healthy controls longitudinally.

Regarding NMDA-receptor antibodies, which is currently the most well-studied antineuronal autoantibody in psychiatric disorders, a meta-analysis found these autoantibodies in the peripheral blood of 8% of patients with schizophrenia [23]. However, antineuronal autoantibodies need to reach the brain to be pathogenic, and currently the prevalence of antineuronal autoantibodies in the CSF of patients with psychotic disorders compared to healthy controls is unknown. A handful of studies has screened the CSF of patients with psychotic disorders for antineuronal autoantibodies [24], with a recently published German study [25] including 456 patients with non-affective psychotic disorders, where they found autoantibodies against surface antigens in the CSF of 0.6%. However, none of them have compared the findings hereof with the CSF of healthy controls, limiting the interpretation of the clinical relevance of the prior findings. A wide array of other antineuronal autoantibodies, such as LGI1 and Caspr2 are also known to be able to induce types of encephalitis with common psychiatric features [19], and the prevalence hereof is even less investigated.

It seems plausible that inflammation is present and immunological pathways are altered in at least a subgroup of patients with psychotic disorders. Identification of this potential subgroup is highly relevant, as it can improve our understanding of the etiological processes involved in psychosis and give rise to a wide range of new treatment options in psychiatry; amongst other the possibility of personalized medicine, and treatment with immunomodulating drugs. To gain more knowledge in this field, and move closer to clinically relevant conclusions, large, systematic, comprehensive studies of both CSF and blood of patients with psychotic disorders and matched healthy controls, with thorough assessment of psychopathology, level of functioning and cognition, analyses of a wide array of neuroimmunological biomarkers, and at least one follow-up visit in order to assess longitudinal changes and correlation with clinical measurements, are needed.

### Aim

We aim to investigate the role of neuroinflammation in patients with psychotic disorders, in order to obtain increased knowledge on the immunological pathways hypothesized to play a role in the development of psychosis in at least a subgroup of patients. With thorough evaluation of clinical measures such as psychopathology and level of functioning, and longitudinal measurements of immunological alterations, we additionally aim to assess the clinical relevance of these neuroimmunological findings.

This will be the hitherto most extensive investigation of immune related changes in CSF and blood from people with psychotic disorders compared to healthy controls, and the first study to assess these changes longitudinally.

## Methods and design

As part of the larger PSYCH-FLAME study, we will investigate neuroimmunological differences in a new cohort of at least 100 adults with recent onset of non-affective psychotic disorders (ICD-10: F20, F22-29), and 100 sex and age matched healthy controls. All clinical data and biological samples from the participants will be saved as part the establishment of a new biobank with focus on research in biological and precision psychiatry at the Mental Health Centre Copenhagen, Denmark. The study was approved by The Regional Committee on Health Research Ethics in the Capital Region of Denmark (De Videnskabsetiske Komiteer) (approval number: H-16030985), September 7, 2016, and February 1, 2019 after funding was obtained. The study was additionally approved by the Danish Data Protection Agency (approval number: RHP-2016-020, I-Suite no.: 04945) on August 31, 2016 and updated July 16, 2018.

### Study design

A prospective case-control study of at least 100 patients with recent onset of a non-affective psychotic disorder (ICD-10: F20, F22-29) and 100 healthy controls matched on sex and age.

### Participants

#### Inclusion and exclusion criteria for cases

Inclusion criteria for cases:

1. Patients with a first-time diagnosis within the past year of a non-affective psychotic disorder (ICD-10: F20, F22-29)
2. Age between 18 and 50 years
3. Obtainment of written informed consent

For exclusion criteria, see Table 1.

**Table 1:** Exclusion criteria.

Exclusion criteria	Rationale
*All participants*	
**1.** Prior psychotic disorders (diagnosis within ICD-10 F20-29)	This study aims to investigate patients with recent onset of non-affective psychotic disorders. Therefore, all potential patients are screened for prior psychotic disorders (i.e. psychotic disorders diagnosed more than one year ago) and excluded if this cannot be ruled out.
**2.** Organic psychiatric disorder	Potential participants with known organic psychiatric disorder or known organic cause to their symptoms (e.g. encephalitis) will be excluded.
**3.** Severe neurological disorder	Potential participants with severe neurological disorders, including severe head injury within the past 3 months, epilepsy (with seizures/possible seizures within the past 10 years), multiple sclerosis, stroke, brain tumor and other disorders with expected or possible impact on the brain’s immune system, are excluded to avoid interference with results from other causes of neuroinflammation.
**4.** Severe general medical conditions	Potential participants with severe general medical conditions involving diseases that have major impact on the immune system including active infection, cancer, autoimmune disorders (e.g. inflammatory bowel disease, multiple sclerosis, or systemic lupus erythematosus), hypothyroidism or hyperthyroidism will be excluded. Participants with known mild asthmatic bronchitis, mild allergies, or other common, mild somatic disorders will be included in both groups to avoid selection bias.
**5.** Contraindications against lumbar puncture	It is urgent to this study that the risk of serious side effects is minimized. Potential participants with contraindication against lumbar puncture will therefore be excluded. Contraindications includes increased risk of bleeding (known International Normalized Ratio (INR) > 1.5, blood platelets < 40, blood thinning treatment or a history of easily getting bruises), signs of increased intracranial pressure (postural headache, recent onset morning headache, nausea) or fever.
**6.** Regular use of anti-inflammatory medication	All participants are screened for use of anti-inflammatory medication including over the counter drugs.
	A potential participant will be excluded if they have a regular (daily or near-daily use of anti-inflammatory medication (including Non-steroidal Anti-inflammatory Drugs (NSAIDs)). The participant can be included after a quarantine period of 14 days if still fulfilling the inclusion criteria.
**7.** Electroconvul-sive therapy (ECT)	ECT in itself is known to induce a proinflammatory response within the brain [26] and current ECT treatment will lead to exclusion. After a 3 months quarantine period the participant can be included if still fulfilling the inclusion criteria.
**8.** Current abuse of alcohol or drugs	Recreational use will be accepted, also among healthy participants, since we aim for the results to be as generalizable as possible. If the use of alcohol or drugs could have caused the current psychiatric illness, the participant has been given an ICD-10 diagnosis of substance abuse (F1) or drugs are used on a daily basis, the potential participant will be excluded.
**9.** Not being able to participate in SCAN-interview	Minor psychiatric co-morbidity will not be an exclusion criterion (e.g. anxiety), but if the participant has psychiatric co-morbidity to an extent that makes the SCAN-interview impossible (e.g. severe autism or mental retardation), he/she will not be included. Participants who cannot participate in a (full) SCAN-interview due to their current psychiatric illness will still be included. The interview can be carried out in Danish or English; participants who due to a language barrier cannot fully participate in our interview will be excluded.
**10.** Pregnancy	As a precautionary principle, pregnant women are not allowed to participate. Pregnancy is known to impact the female body in multiple ways including alterations of the immune system. A risk of pregnancy or known pregnancy leads to exclusion from the study. Lactating women are allowed to participate.
*Healthy controls only*	
**11.** Any current or prior psychiatric disorder	Healthy controls are screened for prior psychiatric disorders. Initially by phone, but also during the SCAN-interview carried out prior to biological sampling; even though the SCAN interview is based on the past 28 days, all participants will be asked if they have ever experienced any of the symptoms. If the interview reveals possible prior psychiatric disorder, the healthy control is excluded.

#### Healthy controls

Inclusion criteria for healthy controls:

1. Age between 18 and 50 years
2. Obtainment of written informed consent

For exclusion criteria, see Table 1.

### Study procedure

**Fig 1:**
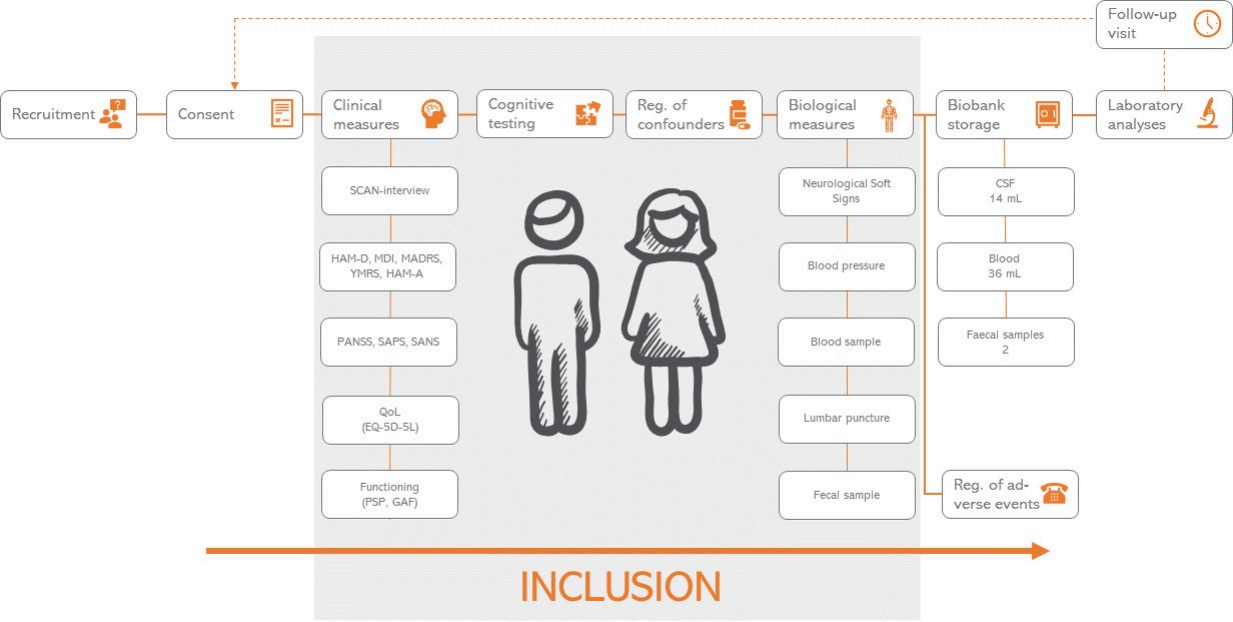
Overview of the study procedure. Patients are recruited from in- and out-patients mental health clinics in the Capital Region of Denmark, healthy controls from internet advertisements and posters. Informed consent is obtained prior to any other intervention procedures. During the intervention, which is sought to be completed in 1 day, information on both clinical and biological measures are obtained, including 44 mL of blood and 16 mL of cerebrospinal fluid (see description of abbreviations below). Cognitive testing is performed using the Brief Assessment of Cognition in Schizophrenia, Mini-Mental State Examination, Montreal Cognitive Assessment and Trail Making Test A and B. Additionally, information on a broad spectrum of possible confounders is obtained, including medication, Body-Mass Index, smoking status and more. A small part of the biological samples is initially analyzed, while the majority will be stored in a biobank for later analyses. All participants are contacted on the day after inclusion for registration of adverse events, and all participants will be invited in for a follow-up visit including the same procedures at least one year later. Abbreviations: HAM-D; Hamilton Depression Scale, MDI; Major Depression Inventory, MADRS; Montgomery-Asberg Depression Rating Scale, YMRS; Young Mania Rating Scale, HAM-A; Hamilton Anxiety Scale, PANSS; Positive And Negative Symptom Scale, SAPS/SANS; Scale for Assessment of Positive/Negative Symptoms, QoL; Quality of Life, PSP; Personal and Social Performance scale, GAF; Global Assessment of Functioning, CSF; Cerebrospinal Fluid.

For an overview of the study procedure, see Fig 1. The study procedure is described in detail in the following.

#### Recruitment and screening

The patients will be recruited via psychiatric departments and outpatient clinics in the Capital Region of Denmark (see S1 Table for an overview of involved centers). Healthy controls will mainly be recruited via a Danish web portal used to find participants for clinical studies (www.forsøgsperson.dk).

Patients will be screened for fulfilment of inclusion and exclusion criteria through their medical journals, including lists of current and prior diagnoses, medical treatment, and blood test results. The healthy controls will be asked a series of screening questions concerning their prior and current mental and physical health, both at first contact via telephone, and on the day of inclusion (see text section 1 in S1 Methods, “Screening questions for healthy controls”). On the day of inclusion, fulfilment of criteria for all participants will be validated through initial screening prior to all other procedures.

#### Intervention

If an eligible participant agrees to be included in the study, written consent will be obtained. For each participant, we aim to perform all clinical measurement and obtain all biological samples on the same day. The inclusion will start between 9 and 10 am. The approximate duration of the inclusion is 3 hours for healthy controls, and 4.5 hours for patients, the latter including a lunch break. At any time during the inclusion, participants can take breaks as needed.

All participants will undergo an interview using WHO Schedules for Clinical Assessment in Neuropsychiatry (SCAN, version 2.1) [27], covering the last 4 weeks. The following chapters were included as they are considered relevant for the included diagnoses; 4 (anxiety), 6 (depressed mood), 7 (thinking, concentration, and energy), 8 (appetite and sleep), 10 (mania), 16 (perceptual disorders), 17 (hallucinations), 18 (thought interference and replacement of will), and 19 (delusions). All interviewers are certified in performing SCAN interviews. For the patients, the interview will be used to confirm diagnoses given by the clinical doctors. Controls will be interviewed prior to the blood sampling and lumbar puncture to ensure that no psychiatric disorders are present, and the time frame for all questions will be extended to include previous possible psychiatric disorders, to ensure that no history hereof exists either.

All participants will be rated on multiple psychopathology rating scales as listed in the following: Positive and Negative Symptom Scale (PANSS) [28], Scale for Assessment of Positive/Negative Symptoms (SAPS/SANS) [29, 30], 17-item Hamilton Depression Rating Scale (HAM-D-17) [31], Montgomery-Asberg Depression Rating Scale (MADRS) [32], Hamilton Anxiety Rating Scale (HAM-A) [33], and Young Mania Rating Scale (YMRS) [34]. Functioning will be rated using Personal and Social Performance Scale (PSP) [35], and Global Assessment of Functioning (GAF) [36].

During the visit, all participants will be asked to complete a questionnaire assessing depressive symptoms, the Major Depression Inventory (MDI) [37], and a questionnaire for the assessment of quality of life and overall health, the EQ-5D-5L [38].

Cognitive testing of all participants will be done using the Brief Assessment of Cognition in Schizophrenia (BACS) [39], Montreal Cognitive Assessment (MoCA) [40], Mini-Mental State Examination (MMSE) [41], and Trail Making Test (TMT) [42] A and B. All testers are certified in using BACS. Additionally, participants are asked to spell the Danish word “klode” backwards and mention as many animals as possible in 1 minute.

All participants will undergo a thorough neurological examination including scoring on the Neurological Evaluation Scale (NES) [43] for detection of neurological soft signs, such as trouble with fine motor coordination and audiovisual integration (for a description of both, see text section 2 and 3 in S1 Methods).

During the visit, all participants will be asked to complete a Danish questionnaire on diet and exercise [44]. Additionally, the following data on possible confounders are registered; height, weight, blood pressure, pulse, smoking status, alcohol intake, use of recreational drugs, allergies, somatic illnesses, prior and concurrent psychiatric disorders, current medication, use of NSAIDs, paracetamol, and antihistamines in the prior 2 weeks, use of antibiotics in the prior 6 months, information on female participants’ menstrual cycle, time for last intake of food and years of education, as well as employment status.

#### Biological samples

Time points for collection of all biological samples will be noted. To minimize diurnal variation in the biomarkers, we aim to collect all blood samples between 9.30 and 11 am, and all CSF samples between 10 and 12 am. For both blood and CSF, a sterile culture swab will be used to perform a swab sample of the skin above the point of needle entry prior to sampling. The swab sample will be stored at -80 degrees Celsius.

The procedures are described below. For further details, see text section 4 in S1 Methods on the collection of blood samples and text section 5 in S1 Methods on the collection of CSF.

Venous blood samples from all participants will be collected prior to the lumbar puncture. A maximum of 44 mL of blood will be sampled.

Lumbar puncture will be carried out according to current consensus guidelines for lumbar puncture [45]. It will be noted whether 1) the participant is placed in a lateral decubitus or sitting position, 2) a traumatic or atraumatic needle is used and 3) whether any anxiolytics (e.g. benzodiazepines) has been taken by the participant prior to the procedure. When possible, we will use lateral decubitus position and atraumatic needles. Lumbar punctures will be performed under sterile conditions, using sterile gloves, sterile covers and face masks. A maximum of 16 mL of CSF will be collected.

As part of the construction of the larger biobank, each participant is asked to provide a fecal sample, either during the visit for inclusion, or afterwards from home. Fecal samples are collected in two ways, both in a Sarstedt Faeces Container and with the OMNIgene Gut Sample Collection Kit. If done at home, the fecal samples will be sent via mail, and stored at -80 degrees Celsius when received at the research unit. Samples collected during the visit will be frozen immediately. The Bristol Stool Scale score, time and date of sampling, as well as time and date of freezing is noted.

#### Follow-up

All included patients will be offered a follow-up visit, which will take place at least one year after the first visit. Controls that matched patients who agreed to a follow-up visit, will also be offered a second visit. Follow-up visits will consist of the same procedures as those mentioned above.

### Laboratory procedures

#### Initial handling and storage

Eight mL of the collected blood and 2 mL of the collected CSF are initially analyzed. Initial blood analyses include white blood cell (WBC) count, differential WBC count, hemoglobin, platelet count, HbA1c, glucose, hs-CRP, albumin, and IgG. Initial CSF analyses include WBC count, differential WBC count, erythrocyte count, protein, glucose, albumin, and IgG. Analyses of cells in CSF and blood are done using Sysmex XN9000, with distribution of cells in the CSF analyzed using CellaVision DM 96. HbA1c is measured using TOSOH G8, albumin and glucose using an ABL800 FLEX radiometer and the rest of the initial CSF and blood analyses are performed on Cobas 8000 module c502 (Roche). We aim to perform all initial CSF analyses and freeze all the remaining CSF no later than 1 hour after sampling. All laboratory personnel will be blinded to all clinical information.

A total of 36 mL of blood and 14 mL of CSF are processed for storage for later analyses, both as droplets on PKU cards, and as small aliquots frozen at -80°C. For details on handling and storage, see text section 6 in S1 Methods on handling of samples.

#### Analyses of stored samples

The initial analyses of the biobank samples stored in small aliquots will be conducted when the first 100 patients with psychotic disorders and 100 healthy controls have been included.

Cytokines and chemokines will be analyzed using the Mesoscale V-PLEX Neuroinflammation Panel 1 Human Kit, by Statens Serum Institut, according to the manufacturer’s instructions.

Antineuronal autoantibodies will be analyzed at Odense University Hospital, with the Euroimmun Mosaic 1 kit (“Autoimmune encephalitis”) combined with the Euroimmun Gad65 Elisa kit, both used according to the manufacturer’s instructions.

All analyses will be performed in a blinded fashion, with no laboratory staff having access to clinical information on participants. Analyses will be performed according to a statistically designed experimental schedule based on a randomized block design or similar, to minimize the risk of confounding by time of analysis, batch, and other factors.

### Outcome measures

#### Biomarkers

Table 2 gives an overview of the below mentioned outcomes.

**Table 2:** Outcomes.

**Primary outcomes**		**Rationale**
Initial screening analyses	CSF WBC	*White blood cell count in CSF is relevant for detecting possible autoimmune encephalitis*, *CNS infections and other types of neuroinflammation*.
	CSF/serum albumin ratio	*Albumin ratio serves as an indicator of BBB permeability*. *Indications of an impaired BBB in patients with psychotic disorders have been found* [18] *and has been suspected to allow entrance of immune cells and cytokines into the CSF*.
	Total protein levels in CSF	*Total protein levels have previously been found to be increased in schizophrenia* [46], *indicating ongoing inflammatory processes*, *and an increased BBB permeability*.
	IgG index	*Increased IgG index reflects IgG production in the CNS*, *indicating possible autoimmune activity*, *and can serve as a proxy for oligoclonal bands*, *used to diagnose multiple sclerosis* [47, 48].
Cytokines and chemokines	CSF levels of IL-6 and IL-8	*Both IL-6 and IL-8 are important pro-inflammatory cytokines*, *involved in multiple immunological pathways*, *and suspected to play a role in schizophrenia* [49]. *CSF IL-6 and IL-8 of patients with psychotic disorders have been found to be elevated compared to healthy controls in a recent meta-analysis* [18].
Antineuronal antibodies	Presence of any antineuronal autoantibody in CSF	*Antineuronal antibodies have been found in peripheral blood of 8% of patients with schizophrenia* [23], *and in the CSF of approximately 1%* [25] *and are known to be able to cause encephalitis with psychotic symptomatology*.
	Presence of any antineuronal autoantibody in CSF or blood	
**Secondary outcomes**		
Initial screening analyses	Neutrophile/lymphocyte ratio (NLR) in blood and CSF	*NLR is increasingly used as an inexpensive measure of systemic inflammation*. *It was found to be increased in the blood of patients with psychotic disorders* [50] *and appears to correlate with clinical outcomes across multiple psychiatric disorders* [51].
	CSF cell type distribution	*Differential count of WBC in psychotic disorders have so far only been assessed in blood of patients*, *and several types of WBC have been found to be increased* [52].
	Blood hs-CRP	*CRP is a non-specific inflammatory marker*, *that has been found to be increased in patients with schizophrenia* [53], *and to be associated with cognitive deficits* [54].
	Blood WBC	*White blood cell count have been found to be increased in schizophrenia* [52] *and is a sign of peripheral inflammation*.
	CSF/serum IgG ratio	*IgG ratio serves as another indicator of BBB permeability*, *and indicates possible intrathecal IgG production*
Cytokines and chemokines	CSF levels of; Eotaxin, Eotaxin-3, IFN-γ, IL-1α, IL-1β, IL-2, IL-4, IL-5, IL-7, IL-10, IL-12/IL-23p40, IL-13, IL-15, IL-16, IL-17A, IP-10, MCP-1, MCP-4, MDC, MIP-1α, MIP-1β, TARC, TNF-α, TNF-β	*Levels of several cytokines have been found to be elevated in plasma compared to controls* [55], *and some appear to be altered by antipsychotic treatment* [56]. *It is reasonable to assume that patterns of several cytokines*, *as opposed to individual ones*, *is involved in the possible inflammatory etiology of some psychotic disorders*.
Antineuronal antibodies	Presence of specific antineuronal autoantibodies in blood and CSF; CASPR2-Ab, LGI1-Ab, NMDAR1-Ab, GABA-B receptor 1, Glutamate receptor 1, Glutamate receptor 2, GAD65	*Specific antineuronal antibodies are known to cause different types of psychiatric symptomatology* [19], *making specific level analyses clinically relevant*.

**Abbreviations:** CSF: cerebrospinal fluid, WBC: white blood cell count, CNS: Central nervous system, BBB: blood-brain barrier, IL: interleukin, IgG: immunoglobulin G, IFN: interferon, IP-10: interferon-gamma induced protein 10, MCP: membrane cofactor protein, MDC: macrophage derived protein, MIP: macrophage inflammatory protein-1, TARC: thymus- and activation-regulated chemokine, TNF: tumor necrosis factor, CNS: central nervous system, NMDA: N-methyl-d-aspartate, AMPA: alpha-amino-3-hydroxy-5-methyl-4-isoxazolepropionic acid, CASPR2: contactin-associated protein 2, LGI1: leucine-rich glioma-inactivated protein 1, GABA: gamma-Aminobutyric acid, GAD65: glutamic acid decarboxylase-65.

The four co-primary outcomes for our initial screening analyses of CSF and blood will be CSF leukocytes, blood/CSF albumin ratio, CSF total protein and IgG index. For cytokine and chemokine CSF levels, IL-6 and IL-8 are the two co-primary outcomes. For antineuronal autoantibodies the two co-primary outcomes will be presence of any one of seven antineuronal autoantibodies (CASPR2-Ab, LGI1-Ab, NMDAR1-Ab, GABA-B receptor 1, Glutamate receptor 1, Glutamate receptor 2, GAD65) in 1) CSF and 2) in either blood or CSF.

As, secondary outcomes, more exploratory analyses will be performed on broader panels of cytokines, specific antineuronal autoantibodies, and other neurological/inflammatory markers. Possible associations between all biomarker outcomes and several clinical measurements will additionally be analyzed.

In our primary publication we will furthermore include reports of adverse events to the lumbar puncture procedure.

#### Measures of safety

In order to assess the risk of adverse events associated with lumbar puncture, participants are contacted by telephone the day after the intervention. Adverse event that could be due to the procedures are noted, with specific focus on possible post lumbar headache and infections.

#### Registry data

Data in the biobank will eventually be linked to the nationwide Danish registers to obtain information on, amongst others, prior infections treated in the primary care sector, comorbidities, treatment outcome (e.g. psychiatric readmissions and change in medication), and later psychiatric diagnoses.

## Power calculation

Power and sample size calculations based on two-sample *t*-tests using standardized mean differences (SMDs) from our prior meta-analysis [57] of CSF studies, show that in comparisons with 100 subjects in each group we are expected to have high power (>99%) for some outcomes (such as CSF/serum albumin ratio, based on an SMD of 0.71), good power (80–85%) for other outcomes (such as total CSF protein, based on an SMD of 0.41) while the power for other outcomes such as CSF WBC count may be as low as 30%. In scenarios where the power with no censoring was 80% or more, simulation analyses showed that censoring rates up to 33% and 50% decreased the power using a censored Gaussian model by at most 5%- and 10%-points respectively compared to no censoring.

### Statistical models/Analysis

Possible differences in demographic data will be examined using two-sample *t*-tests when outcome is continuous, and χ^2^ test when categorical (e.g. sex).

Possible differences in continuous variables (such as level of IL-6 or IgG index) between patients and healthy controls will be analyzed using linear models with group, age, and sex as covariates. Outcomes measured either as concentrations (e.g. IL-6) or ratios (e.g. CSF/serum albumin ratio) will be log-transformed prior to analyses. Associations between levels of biomarkers and clinical measures such as psychopathology (total PANSS score, SANS/SAPS score), cognitive measures, medication status, duration of illness, and level of functioning will also be investigated. Differences between patients and healthy controls of dichotomous variables (such as presence of autoantibodies, yes/no) will be analyzed using Pearson chi-square tests. Two-sided tests with p<0.05 will be considered significant. If a continuous outcome variable has measurements censored below the lower limit of quantification a censored Gaussian model will be used instead of linear models

All statistical analyses will be performed in R [58]. For the censored Gaussian model the package “survival” will be used [59].

### Plan for publication and future data collection

The first publications will follow the inclusion of the first 100 participants with recent debut of non-affective psychosis and 100 sex and age-matched controls. This will initially result in three publications; one comprising all initial screening analyses, one investigating all cytokines and chemokines and one on antineuronal autoantibodies (see Table 2 and outcome measures above). Inclusion of participants, including follow-up visits, will continue, resulting in the construction of a larger cohort and biobank. Future analyses on this larger cohort and biobank will be thoroughly described in future separate protocols, as the biobank continues to grow.

All data will be stored in a secure manner in the REDCap electronic data capture tools [60, 61] hosted at the Capital Region of Denmark, as well as in paper forms in archive lockers, to ensure future use of the data. Sufficient meta data to fully understand the saved data is additionally saved in secured folders.

## Discussion

### Strengths and limitations

The project described in this report is part of the larger PSYCH-FLAME project investigating the immune system’s involvement in psychotic and affective disorders, from a nationwide angle with large-scale register-based studies, immunogenetic studies, and through CSF and blood biobank studies, acting in synergy to advance the immunopsychiatric field. The clinical study described in this protocol on recent onset psychosis and sex- and age-matched healthy controls will include a large number of participants compared to previous studies, increasing our power and thereby probability of detecting relevant biomarkers. Our intervention comprises a broad and comprehensive assessment of psychopathology, level of functioning, quality of life, cognitive functioning, and many possible confounders. We take into account possible diurnal variations in immunological biomarkers, by performing all lumbar punctures and blood samples in as narrow a time frame as possible, and by thoroughly registering time points for collection of blood, CSF and feces. We register medical treatment, both psychiatric and other, via electronic health records and self-report. We register height, weight, smoking habits, alcohol use, diet, and exercise habits of all participants, allowing for the control of these possible confounders’ influence on the measured biomarkers. In general, we have assured that we completely fulfill all criteria for a very high quality study, minimizing risk of bias according to the Newcastle Ottawa Scale [62].

#### Case assessment and definition

Previous studies have found an increased peripheral inflammatory response in patients with psychotic disorders, particularly during the first psychotic episode [11, 18]. In order to investigate if also neuroinflammation is present in these patients, we only include patients with recent debut of the psychotic illness. Thus, the case group is also more homogenous making the patients more comparable.

To ensure an accurate case definition, we will independently, through our comprehensive SCAN-interview, validate the patients’ diagnoses given by the clinicians. WHO’s SCAN-interview is a broadly tested and widely used instrument in assessment of psychopathology [27]. In our interview we mainly focus on anxiety, depressive, manic and psychotic symptoms. This raises the possibility of not diagnosing other concurrent psychiatric disorders, such as ADHD or eating disorders, but we chose to do so to limit the time needed for the interview and make it more bearable even to the most severely ill patients. Furthermore, with the chosen chapters of the SCAN-interview we are certain that we can reliably validate the diagnoses within the psychotic spectrum.

Our follow up visit and the possibility of linking data to Danish registers, further makes it possible for us to, over time, validate the diagnoses given.

#### Control assessment and definition

An important strength of our study is our healthy control group, as only few previous studies investigating immunological alterations in the CSF of patients with psychotic disorders have included a group of healthy controls. We thoroughly assess possible psychopathology in our healthy controls before including them in our study and interview them using the SCAN-interview just as we do with our cases. This allows us to exclude any subjects with current or previous psychiatric disorders. Additionally, many of the symptoms occurring in our patients might also to some degree be present in otherwise completely healthy controls (such as increased/decreased appetite for a period of time, issues with sleep, or some level of concentration difficulties). With our thorough SCAN-interview and multiple rating scales, we can adequately evaluate the severity of any possible symptoms. Recruiting healthy volunteers from the website forsøgsperson.dk allows us to include individuals from the same broad community as the cases.

#### Risk of bias

When including a vulnerable group of patients, a risk of selection bias is difficult to avoid. The most severely ill patients might be less likely to agree to participate, particularly since the intervention is somewhat comprehensive. To overcome this to the greatest possible extent, we have tried to keep our intervention as brief as possible, whilst including all relevant variables, and have managed to limit the duration of the intervention to a single day. When recruiting patients, we will strongly emphasize the possibility of multiple breaks, and that the patients may leave at any time, should they feel the need to stop the intervention. Additionally, we arrange and pay for transport via taxi to and from the inclusion, and for those who are admitted to hospital wards, we offer to perform the intervention at their ward. Another possibility of selection bias stems from our strict inclusion and exclusion criteria. However, the nature of the study, investigating multiple biomarkers that broadly represent many aspects of the immune system, establishes the need for stringent exclusion of other factors with impact hereon.

Some confounders such as smoking, alcohol use, and eating habits, rely on self-reported data, raising the issue of possible recall bias, and limits the validity of some of these data.

### Risks and side effects

The potential side effects of this study are limited to adverse events following the procedures during the sampling of blood and CSF, since medical treatment is not included in the trial. All participants will be given information on how to contact the doctors responsible for PSYCH-FLAME in case any adverse events occur, and to thoroughly assess these possible adverse events, participants will furthermore be contacted via telephone by one of our doctors on the day after inclusion.

The risk of infection associated with the procedure of a common blood test is very low and the risk of infection after a lumbar puncture is considered extremely small, with some authors estimating an incidence of less than 0.01% [63]. The blood volume removed represents approximately 1% of the total blood volume, which does not result in any physiological consequences. The average adult is estimated to have a total CSF volume of approximately 150 mL, which is replaced 4–5 times per day [64]. During our lumbar puncture procedure, we will sample around 10% of the volume, which might result in slight dizziness in the minutes following the procedure. However, due to the fast production of CSF, the removed volume will be replaced in 20–30 minutes. The risk of post lumbar headache has been shown to be around 4% when using atraumatic needles, which will be the most commonly used needle in the present study [65]. Post lumbar headache is a benign, most commonly self-limiting, condition, that can be handled with simple painkillers, increased intake of liquid, and rest. If headache persists, the participant can be referred either to a blood patch, a well-known, simple and safe procedure [66], or to the newer even less invasive procedure of a sphenopalatine ganglion block [67].

#### Unexpected findings

Some of the clinical examinations and biological samples will result in unexpected findings regarding the health status of patients or healthy controls. If a consent to be contacted regarding such findings has been given, the participant will be contacted and referred to further relevant investigations (e.g., at a neurological department). If the findings are found to be severe or life threatening, the participant will be contacted regardless of consent.

### Ethical considerations

The study will be conducted in accordance with the principles of the Declaration of Helsinki (64thWMA general assembly; Fortaleza, Brazil, October 2013), and other applicable laws and regulations. Participation in the study is preceded by elaborate information on the entire course of the study, potential individual benefits and personal risks. It is emphasized that participation is absolutely voluntary. Patients are given sufficient time to read all provided information, and no procedures will take place before written consent is obtained. This consent can be revoked at any time. Before the lumbar puncture is performed, all participants are investigated for contraindications. All individuals will be anonymized when analyzing the data. The identity of participants will only be used for merging of the data and retrieving the biological samples. Data will only be published in a manner where no individuals can be identified.

### Perspectives and clinical implications

This study and the PSYCH-FLAME biobank construction will represent the largest, most comprehensive study to date on immunological alterations and differences in levels of neurological biomarkers in the CSF between patients with psychotic disorders and healthy controls. Additionally, it will be the first time that biomarkers in the CSF of patients with psychosis and matching healthy controls are assessed longitudinally. This will provide unique insight into possible correlations between clinical status and neuroinflammation. A substantial part of patients with psychotic disorders experience low treatment response of current antipsychotic treatment, and psychotic disorders have huge impact on the patients’ functioning and quality of life, causing suffering on both themselves and relatives, while bearing great costs to society [1]. The identification of a subgroup of patients with immunological alterations or presence of antineuronal autoantibodies will not only provide insight into possible etiological mechanisms, but will also pave the way for the investigation of treatment with for example immunomodulating drugs based on biomarkers, which our research group will subsequently pursue to test in RCTs with anti-inflammatory treatment based on our results. This would be an important and ground-breaking step towards introducing precision medicine in psychiatry.

## Study status

The study is ongoing. Participants are currently being recruited, and inclusion for this first part is expected until August 1, 2021, after which the first analysis described in this protocol will initiate, and inclusion will continue hereafter in order to build a larger biobank.

## Declarations

### Ethical approval and consent to participate

The study is approved by The Regional Committee on Health Research Ethics (Capital Region, j.no: H-16030985) and The Danish Data Protection Agency (j.no: RHP-2016-020, I-Suite no.: 04945).

Before any collection of data, participants will receive thorough information about the study (implications, procedures and possible adverse events), both orally and in writing. All participants will be given sufficient time to consider participation before consent is given. The patient can at any time retract their consent and will then no longer be part of the study unless the material has already been analyzed. This will have no influence on their further treatment in the psychiatric department/clinic.

### Identification and pseudoanonymization of patient data

All participants will be identified using their unique Civil Registration Number which is assigned to all Danish citizens at birth or at the time of immigration. When informed consent has been given, participants will be registered by their Civil Registration Number in an identification key kept separately from all other data, and data will be pseudoanonymized by a study ID-number, as according to the approval from the Danish Data Protection Agency. This will make it possible to later expand the project with data from the many different Danish registers.

## Supporting information

S1 Table Centers involved in the recruitment of patients with psychotic disorders. (DOCX)

S1 Methods Supplementary methodological descriptions. (DOCX)
