# Supplementary material for: Immunological investigations of the cerebrospinal fluid in patients with recent onset psychotic disorders: A study protocol
Source: PLoS One. 2021 Sep 29;16(9):e0257946. doi: 10.1371/journal.pone.0257946 (PMC8480791; doi:10.1371/journal.pone.0257946)
Supplement: S1 Table — (DOCX) [file pone.0257946.s001.docx]

## S1 Table: Centers involved in the recruitment of patients with psychotic disorders

| **Mental Health Services in the Capital Region of Denmark** |
| --- |
| ***In-patient units of the Mental Health Centers (in the Capital Region of Denmark)*** |
| Mental Health Centre Copenhagen |
| *Rigshospitalet*  *Bispebjerg Hospital*  *Gentofte Hospital*  *Frederiksberg Hospital* |
| Mental Health Centre Amager |
| Mental Health Centre Ballerup |
| Mental Health Centre Glostrup |
| *Glostrup Hospital*  *Hvidovre Hospital* |
|  |
| ***Out-patient mental health clinics (in the Capital Region of Denmark)*** |
| OPUS teams (early detection and intensive case management of young patients with psychosis) |
| *OPUS Nørrebro*  *OPUS Indre by/Østerbro*  *OPUS Frederiksberg*  *OPUS Amager*  *OPUS Ballerup*  *OPUS Hvidovre/Valby*  *OPUS Glostrup* |

# 
