# Supplementary material for: Immunological investigations of the cerebrospinal fluid in patients with recent onset psychotic disorders: A study protocol
Source: PLoS One. 2021 Sep 29;16(9):e0257946. doi: 10.1371/journal.pone.0257946 (PMC8480791; doi:10.1371/journal.pone.0257946)
Supplement: S1 Methods — (DOCX) [file pone.0257946.s002.docx]

# S1 Methods: Supplementary methodological descriptions

## Table of contents

1. Screening questions for healthy controls (page 1)
2. Neurological examination (page 1)
3. Performance and scoring on Neurological Evaluation Scale (page 2)
4. Collection of blood samples (page 6)
5. Collection of CSF (page 7)
6. Handling of samples (page 8)

## 1. Screening questions for healthy controls

- Do you consider yourself healthy?
- Do you suffer from any physical illnesses?
- Do you suffer, or have you previously suffered, from any psychiatric disorders, such as eating disorders, anxiety, depression, OCD, ADHD or others?
- Have you previously been in contact with a psychologist or psychiatrist?
- Do you currently take any medication?
- Have you previously, for a longer period of time, taken any medication?
- Do you have any allergies?

## 2. Neurological examination

- Assessment of mental status and higher cognitive functioning
  - Consciousness (GCS), orientation (time, place, personal data), aphasia, dysarthria, neglect
- Cranial nerves
  - II; visual field, III+IV+VI; eye movements and pupil function, V; facial sensibility, VII; facial movement, VIII; hearing of finger-rubbing, IX+X; palate movement, XI; head turning and strength of shoulder lift, XII; tongue position and movement
- Motor system
  - Muscle tone, muscle strength in all 4 extremities (both proximal and distal), test of pronator drift, assessment of atrophy, assessment of involuntary movements such as fasciculations,
- Sensory system
  - Light touch on all 4 extremities, Romberg’s test, graphesthesia, stereognosis, extinction
- Reflexes
  - Biceps, triceps, brachioradialis, knee, ankle and plantar response (Babinski), glabellar tap, suck and snout reflex, grasp reflex
- Coordination and assessment of cerebellar function
  - Finger-nose test, assessment of walking, dysdiadochokinesis, ataxia, nystagmus
- Assessment of intracranial pressure
  - Ophthalmoscopy, neck stiffness

## 3. Performance of and scoring on Neurological Evaluation Scale

Participants are scored on the following subcategories; Primary signs (P), sensory integration (SI), motor coordination (MC) and sequencing (SQ).

The tests performed, including their scores, are described below.

- **Smooth Pursuit (P)**

Fixate fingers in near field moved between extremes of lateral vision in approximately 30 secs in both directions twice.

- - **Scoring:** 0 = no noticeable interruption movement; 1 = Minor interruption of smooth movement; 2 = Broken up jerky pursuit.
- **Synkinesis (P)**

Subject is instructed to follow the cap of a pen with his/her eyes only as it is moved between extremes of horizontal gaze. If the subject moves his/her head, the subject is asked to keep his/her head still and follow the cap of a pen with the eyes only.

- - **Scoring (right and left):** 0 = No movement of the head; 1 = Movement of the head on first trial but not when specifically told to keep head still; 2 = Movement of the head even when told to keep the head still.
- **Convergence (P)**

Subject is instructed to follow the cap of a pen with his/her eyes as it is moved toward the subject’s nose.

- - **Scoring (right and left):** 0 = Both eyes converge on object; 1 = One or both eyes are unable to converge completely but converge more than halfway; 2 = One or both eyes fail to converge more than halfway.
- **Gaze impersistence (P)**

Subject is instructed to fix his/her gaze on the cap of a pen at a 45 angle in the horizontal plane of the right and left visual fields for 30 seconds.

- - **Scoring (right and left):** 0 = No deviation from fixation; 1 = Deviation from fixation after 20 seconds; 2 = Deviation from fixation before 20 seconds.
- **Saccade to target (P)**

Subject asked to fixate examiners nose at confrontation, then to look toward right and left hand held up vertically.

- - **Scoring:** 0 = Single smooth rapid movement to target; 1 = More than one movement or low velocity (say which); 2 = more than 2 movements or very low velocity.
- **Saccade to command (P)**

Subject asked to look towards left and look towards right from mid position.

- - **Scoring:** 0 = Single smooth rapid movement; 1 = more than one movement or low velocity; 2 = more than 2 movements or very low velocity.
- **Tone increase (P)**

Assessment of tone during elbow flexion/extension, elbow supination/pronation, and wrist flexion/extension.

- - **Scoring (right and left):** 0 = Normal tone; 1 = Mild increase; 2 = Marked increase, say lead pipe/other.
- **Glabellar reflex (P)**

Subject is instructed to fix his/her gaze on a point across the room. The subject is approached from above the forehead outside the visual field, and the examiner taps the glabellar region 10 times with the index finger.

- - **Scoring**: 0 = 3 or fewer blinks; 1 = 4 or 5 full blinks, or more than 6 partial or full blinks; 2 = 6 or more full blinks.
- **Snout reflex (P)**

Subject is instructed to relax, and the examiner taps vertical finger held against subject'’ philtrum.

- - **Scoring**: 0 = no contraction of the orbicular orris (or puckering of the lips); 2 = any contraction of the orbicular orris (or puckering of the lips).
- **Suck reflex (P)**

The examiner places the knuckle of a flexed index finger or tongue depressor between the subject’s lips.

- - **Scoring**: 0 = no movement; 2 = any pursuing or sucking motion by the subject’s lips.
- **Grasp reflex (P)**

The subject is instructed not to grab, and the examiner strokes the inside of the subject’s palm, between the index finger and the thumb.

- - **Scoring (right and left)**: 0 = no flexion of the subject’s finger; 1 = mild flexion of the subject’s finger on first trial or flexion of any kind on second trial; 2 = marked flexion of the subject’s fingers on first trial.
- **Stereognosis (SI)**

Subject, with eyes closed, is asked to identify an object placed in his/her hand. Subject is instructed to feel the object, he/she is asked to describe for what purpose the object is used. The subject starts with the dominant hand, based on prior evaluation of handedness, or the hand with which he/she writes, if there is mixed hand dominance. The instructions are repeated at the beginning of the second trial.

- - **Scoring (right and left)**: 0 = no errors; 1 = one error; 2 = two or more errors
- **Graphaesthesia (SI)**

Subject, with eyes closed, is asked to identify the number written on his palm. The order of hands is determined as with stereognosis. 3 trials each hand.

- - **Scoring (right and left)**: 0 = no errors; 1 = 1 error; 2 = more than 1 error.
- **Extinction (SI)**

The subject is seated, with hands resting palms down, on his/her knees and with eyes closed. The subject is told that he/she will be touched on either the cheek, hand, or both and is to say where he/she has been touched. If the subject names just one touch, her/she is asked – the first time this occurs only – if he/she felt a touch anywhere else. The simultaneous touching is done in the following order, right cheek-left hand, left cheek-right hand, right cheek-right hand, left cheek-left hand, both hands, both cheeks.

- - **Scoring**: 0 = no errors; 1 = 1 error; 2 = more than 1 error.
- **Right/Left confusion (SI)**

Subject is asked to point to his/her right foot, left hand, place his/her right hand to left shoulder, left hand to right ear, point to examiner’s left knee, right elbow, with examiner’s arms crossed, point to examiner’s left hand with his/her right hand, and with examiner recrossing arms point to examiner’s right hand with his/her left hand.

- - **Scoring**: 0 = no error; 1 = 1 error; 2 = 2 or more errors.
- **Finger-thumb opposition (MC)**

Ask the subject to place both hands palm up with fingers fully extended on his/her legs. The subject is to start with his/her dominant hand and is to touch the tip of his/her fingers with the tip of his/her thumb, from forefinger to pinky, returning to forefinger, for a total of 10 repetitions.

- - **Scoring (right and left)**: 0 = no major disruption of motion and no more than one mistake; 1 = no major disruption of motion or two to three mistakes; 2 = major disruption of motion or four or more mistakes.
- **Mirror movements (P)**

The subject’s hand, which is not performing the Finger-Thumb Opposition Test, is observed for parallel movements of the fingers and thumb.

- - **Scoring (right and left):** 0 = No observable movements of the fingers; 1 = Minor, inconsistent or repetitive movements of the fingers; 2 = Consistent, distinctive movements of the fingers.
- **Rapid alternating movements (MC)**

Ask the subject to place his/her hands palm down on legs. The subject is to start with his/her dominant hand is to slap his/her leg distinctly with the palm and the back of his/her hand in alternating motion. The determination of dominance is described above. The subject is to perform the task 20 times, with both hands, one at a time.

- - **Scoring (right and left)**: 0 = no major disruption of motion, hesitation, or mistake in hand placement; 1 = no major disruption of motion or one to two hesitations or mistakes in hand placement; 2 = major disruption of motion or three or more hesitations or mistakes in hand placement.
- **Finger-Nose test (MC)**

The subject is instructed to close eyes and touch the tip of his/her nose with the tip of his/her index finger.

- - **Scoring (right and left)**: 0 = no intention tremor or passpointing; 1 = mild intention tremor or passpointing; 2 = marked intention tremor or passpointing.
- **Romberg (P)**

Subject to stand with his/her feet together, eyes closed, his/her arms held parallel to the floor, and fingers spread apart. The subject is to maintain this position for 1 min.

- - **Scoring:** 0 = relatively stable, minimal swaying; 1 = marked swaying; 2 = subject steps to maintain balance or falls.
- **Adventitious Overflow (P)**

Same as Romberg Test

- - **Scoring (right and left):** 0 =absence of movement of fingers, hands, or arms; 1= irregular fluttering movement of fingers only; 2 = irregular fluttering movement extended to hands and/or arms.
- **Tremor (P)**

Same as Romberg Test

- - **Scoring (right and left):** 0 = no tremor; 1 = mild, fine tremor; 2 = marked, fine or coarse tremor.
- **Tandem walk (MC)**

Subject to walk, in a straight line, 12 feet, heel to toe.

- - **Scoring**: 0 = no missteps after subject has completed first full step; 1 = one or two missteps after completion of first full steps; 2 = 3 or more missteps, grabbing or falling.
- **Limb hypereflexia (P)**

Assessment of tendon jerks (biceps, triceps, brachioradialis, patella, ancle)

- - **Scoring (right and left):** 0 = Normal; 1 = Mild increase reflexes; 2 = Marked increase reflexes (say if clonus).
- **Babinski (P)**

Assessment of Babinski

- - **Scoring (right and left):** 0 = Big toe flexes; 1 = Equal response; 2 = Big toe extends (not withdrawal response).
- **Audio-Visual integration (SI)**

The subject is asked to match a set of tapping sounds with one of three sets of dots presented on a 5-inch x 7-inch index card. The subject is instructed to close his/her eyes during the tapping. Three practice trials are performed first to ensure that the subject understands the directions.

- - **Scoring**: 0 = no error; 1 = 1 error; 2 = two or more errors.
- **Ozeretski test (SQ)**

The subject is to place both hands on the table, one hand palm down and the other hand in the shape of a fist. The subject is then asked simultaneously to alternate the position of his/her hands in a smooth and steady motion. The subject is asked to repeat this motion 15 times.

- - **Scoring**: 0 = no major disruption of motion after first repetition, errors limited to no more than two hesitancies in the transition from one position to the next and no more than one mistake in hand position. 1 = no major disruption of motion after first repetition or complete breakdown of motion, more than two hesitancies in the transition from one position to another, difficulty in developing and maintaining a smooth and steady flow of movement, three to four position confusion, or any total of three or four errors; 2 = major disruption of movement or complete breakdown of motion or more than four hesitancies or position confusions.
- **Fist-Edge-Palm test (SQ)**

Ask the subject, using a smooth and steady rhythmic pattern, to touch the table with the side of his/her fist, the edge of his/her hand, and the palm of his/her hand. The subject is to break contact with the surface of the table between each change in hand position, but not to bring the arm back in full extension. The subject is to repeat this sequence of position changes 15 times.

- - **Scoring (right and left)**: 0 = no major disruption of motion after first repetition, errors limited to no more than two hesitancies in the transition from one position to the next and no more than one mistake in hand position. 1 = no major disruption of motion after first repetition or complete breakdown of motion, more than two hesitancies in the transition from one position to another, difficulty in developing and maintaining a smooth and steady flow of movement, three to four position confusion, or any total of three or four errors; 2 = major disruption of movement or complete breakdown of motion or more than four hesitancies or position confusions.
- **Fist-ring test (SQ)**

The subject is asked to alternate placing his/her hand on the table, in the position of a fist, with the thumb placed either over the knuckles or over the middle phalanges and placing his/her hand, on the table, in the position of a ring, with the tips of the thumb and forefinger touching and the remaining three fingers extended. The subject is to bring his/her arm into the upright position between each change in hand position. If the subject does not perform the movement accurately or in a manner that can be appropriately assessed, he/she is to be stopped, to be reinstructed, and to start the test again. The subject is to repeat each set of hand position changes 15 times.

- - **Scoring (right and left)**: 0 = no major disruption of motion after first repetition, errors limited to incomplete extension of fingers in ring position and no more than two hesitancies in the transition from fist to ring, and vice versa and no more than one fist/ring confusion. 1 = no major disruption of motion after first repetition or complete breakdown of motion, more than two hesitancies in the transition from fist to ring, difficulty in developing and maintaining a smooth and steady flow of movement, three to four fist/ring confusion, or any total of three but not more than four errors; 2 = major disruption of movement or complete breakdown of motion or more than four fist/ring hesitancies or confusions.

## 4. Collection of blood samples

1. The skin is cleaned by an 82% ethanol/0,5% chlorhexidine swap (Alkoholswabs, Mediq Danmark, Brøndbyvester, Denmark).
2. Using a sterile culture swab (BD BBL CultureSwab, BD BBL, Brescia Italy), we perform a swab sample of the skin above the point of needle entry prior to the sample being taken.
3. A SAFETY Blood Collection Set + Holder 21G x ¾’’ (Greiner Bio-One Gmbh, Kreimsmünster, Austria) needle is used for the collection of the blood sample.
4. The following samples are taken in the below mentioned glasses (Vacuette® Tube, Kremsmünster, Austria):
   1. 1 x 4 mL Lithium Heparin Sep
   2. 1 x 2 mL FX Sodium Fluroide/Potassium Oxalata
   3. 2 x 2 mL K2E K2EDTA
   4. 3 x 6 mL K3E K3EDTA
   5. 3 x 6 mL Z serum Clot Activator
5. Blood samples are stored at room temperature until all CSF samples are available, in order for these to be transported collectively to the University hospital of Copenhagen for handling and analysis.

Whenever anything deviates from the abovementioned protocol, all details hereon will be noted.

## 5. Collection of cerebrospinal fluid

1. The patient is placed in lateral decubitus position.
2. To ensure sterility of the procedure, a sterile area will be prepared on a suitable table with a Barrier® sterile cover (Barrier, Göteborg, Sweden).
3. The examiner will put on sterile gloves (ProFeel® DHD™ Micro Powder Free Latex Surgical Gloves, WRP, Vienna, Austria).
4. The skin area covering the space between the preferred point of needle entry (either between spinous processes L3/L4 or L4/L5) is cleaned twice by using 0.5% chlorhexidin-digluconat/83% v/v ethanol (Region Hovedstadens Apotek, Herlev, Denmark). The liquid is applied by using a sterile Dressing set (Bastos Viegas, Gullhufe, Portugal) starting over the preferred point of needle entry and moving outwards in a spiral.
5. Using a sterile culture swab (BD BBL CultureSwab, BD BBL, Brescia Italy), we perform a swab sample of the skin above the point of needle entry prior to the sample being taken.
6. A Barrier® sterile cover (Barrier, Göteborg, Sweden) with a hole in the center is attached to the patient leaving a sterile area for the lumbar puncture.
7. Local anesthesia will be given subcutaneously at the preferred point of needle entry in the form of 2-4 mL of Lidocaine with a 23G cannula (KDM® KD-FINE®, Berlin, Germany) attached to a 2.5 ml syringe in a sterile procedure.
8. The lumbar puncture will be carried out using an atraumatic 22G needle (RapID™ Spinal Needle Set Pencil Point Spinal Needle, Smiths Medical International Ltd., CT21, 6JL, UK).
9. The first drops of CSF will be discarded, in order to minimize the amount of blood in the samples.
10. The following samples are collected
    1. 2 x 1 mL of CSF is collected in two 5 mL tubes (Sarstedt, Nümbrecht, Germany)
    2. The following 14 mL of CSF will be collected in a suitable 15 mL non-pyrogenic tube (SARSTEDT, Nümbrecht, Germany).
11. As soon as the CSF is collected, it will be transported by taxa together with the blood sample. Routine analyses will be carried out within a maximum of an hour from the collection of the first drop of CSF. The time from lumbar puncture to analysis is noted. Samples are kept at room temperature during transport.
12. The skin is covered with an adhesive surgical dressing (evercare®, OneMed, Helsinki, Finland).

Whenever anything deviates from the abovementioned protocol, all details hereon will be noted (e.g. the usage of a different needle size due to larger BMI of the participant, sitting position instead of lateral decubitus position etc.).

## 6. Handling of samples

All routine analyses have been performed at the University Hospital of Copenhagen. Up until participant number 70, samples were prepared for storage at Statens Serum Institute (SSI). Going forward, this preparation has been performed in an identical manner at the University Hospital of Copenhagen. Transportation time to SSI and the University Hospital of Copenhagen using taxi is similar.

The following describes the handling of the blood and CSF samples that are not used for initial analyses but stored in the biobank.

1. Time of sample arrival, procedure start, and sample ID are noted.
2. The laboratory technician puts on gloves.
3. PKU cards (filter papers, Schleicher & Schuell, Dassel, Germany) are marked with Psych-Flame ID, date and sample type (CSF, Serum or EDTA, before or after centrifugation).
4. 2 x 100 µL of blood from both the Z serum Clot Activator or the K3EDTA container, or CSF, are placed on individual PKU cards marked as “before centrifugation”.
5. All samples are centrifuged at 22ºC at 1145 G for 10 minutes with standard acceleration and deceleration.
6. Step 4 is repeated for PKU cards marked as “after centrifugation”.
7. The PKU cards are left to dry at room temperature overnight, and then moved to -80°C.
8. 1 mL Nunc Cryo Vials (Bio-Greiner) are marked as either Serum, EDTA or CSF.
9. The supernatants of the blood from either the Z serum Clot Activator or the K3EDTA container, or CSF, are pipetted into 500 µL aliquots in separate Nunc Cryo Vials.
10. The samples (including the pellet of the 2 Z serum clot activator tubes, 2 K3EDTA tubes and the CSF tube) are moved directly to storage at -80ºC.
